# Along came a spider: an unusual organism identified in a peritoneal dialysis patient, a case report and literature review

**DOI:** 10.1186/s12882-020-02099-8

**Published:** 2020-11-11

**Authors:** Victoria Jane Carnall, Stephanie Murdock, Cressida Auckland, Christopher J. Mulgrew

**Affiliations:** 1 Exeter Kidney Unit, Royal Devon and Exeter Foundation Trust, Exeter, Devon UK; 2 Microbiology Department, Royal Devon and Exeter Foundation Trust, Exeter, Devon UK

**Keywords:** Peritonitis, Pets, Fungal infection, Rhodotorula, Zoonotic, Case report

## Abstract

**Background:**

Peritoneal dialysis-associated peritonitis can uncommonly be caused by fungal infections. When they do present, they are associated with significant mortality and morbidity. We describe a case where a sample of peritoneal dialysate fluid grew *Rhodotorula muciliginosa*, a yeast organism present in the normal environment which has previously been reported as rarely causing peritonitis. We believe this is the first case where the *Rhodotorula spp.* and its origin has been identified.

**Case presentation:**

A 20 year old male grew *Rhodotorula muciliginosa* from his peritoneal dialysis fluid on three separate occasions when a fluid sample was sent following a disconnection and subsequent set change. He was not systemically unwell and his peritoneal dialysate was clear. As *Rhodotorula spp.* is exceedingly difficult to treat our patient had his Tenchkoff catheter removed. Subsequent samples of soil and sand from his bearded dragon and Chilean tarantula cases, kept in his bedroom where dialysis occurred, were tested. The tarantula sand was identified as the source of the *Rhodotorula spp.* Of note, *Candida* was isolated from sand from the bearded dragon case. Once his Tenchkoff was removed he was treated with an intravenous course of antifungal therapy. He has since had a new Tenchkoff catheter inserted and recommenced PD following education around pets and hygiene.

**Conclusions:**

In this era where people are keeping increasingly rare and unusual wildlife in their homes, this case highlights the need for clinician and nursing staff awareness of a patient’s home environment and hobbies when they are undergoing peritoneal dialysis. Sand from our patient’s tarantula case grew the colonising organism but interestingly soil from his bearded dragon case also isolated candida. This can also cause difficult to treat peritonitis.

## Background

Fungal infections are an uncommon but serious cause of PD (Peritoneal Dialysis)-associated peritonitis [1]. The incidence worldwide is heterogenous with rates varying from 2 to 23.8% [2]. In the United Kingdom an audit study reported a fungal peritonitis rate of 0.0099/patient year from centres not using prophylaxis and a lower rate of 0.0032/patient year in centres using daily oral fluconazole [3]. Despite its rarity fungal peritonitis is important due to its associated higher rates of catheter loss, morbidity and mortality [3]. *Candida* species are the commonest pathogen isolated in fungal peritonitis, accounting for 70–90% of the cases described [3]. Patients at particular risk for fungal peritonitis are those who have had previous bacterial peritonitis, been on prolonged antibiotic treatment, have gynaecological or bowel sources of infection, those who are immunosuppressed, have diabetes mellitus, are malnourished, or have a prolonged time on PD [2, 3]. We describe a case where *Rhodotorula muciliginosa* was isolated from a patients’ peritoneal dialysate fluid following an accidental set disconnection. This organism is a pigmented yeast, previously regarded as an environmental organism of little pathogenic significance, often present on the skin as well as in sputum, urine and faeces [4]. In the environment *Rhodotorula spp.* favours warm, wet locations, such as shower curtains and toothbrushes [5]. Increasing evidence suggests its emergence as an opportunistic pathogen, particularly in the immunocompromised population. Published reports indicate *Rhodotorula spp*. as the likely causative agent in catheter-associated fungaemia and peritonitis, most relevant to our renal patients [6, 7], but also other infections such as meningitis, endocarditis, and prosthetic joint infections [4]. *Rhodotorula spp.* causing PD peritonitis has been reported in eight papers, none of which have identified the source of the organism. One paper in 1983 identified a point source outbreak of three cases in Canada attributable to *Rhodotorula muciliginosa* isolated from environmental sampling, all three patients had their catheters removed [8]. A paediatric patient on APD (Automated Peritoneal Dialysis) had *Rhodotorula muciliginosa* isolated from their dialysate fluid, they were treated with IV antifungal therapy and tube removal [9]. Five adult cases have been reported; one patient punctured his PD bag and continued to use it, he was successfully treated with antifungals. Three further patients had antifungal treatment and subsequent catheter removal [7, 10–12]. The final patient had antifungal treatment and catheter exchange [13]. Unal et al.analysed the clinical presentations, aetiology and treatment of fungal peritonitis in their hospital. Only one case of *Rhodotorula muciliginosa* was identified in their case report of 21 cases. Their patient was treated with amphotericin and the catheter removed [14]. Most reported cases of *Rhodotorula* spp. infections are due to *muciliginosa,* with an incidence of 68.6% reported in a systematic review by Ioannou et in 2018, with *R.glutinis* and *R.minuta* being less common [15]. Our case is the first which has grown *Rhodotorula* spp. from peritoneal dialysate fluid as well as identifying the likely source.

## Case presentation

A 20 year old man with chronic kidney disease stage 5 secondary to chronic interstitial nephritis had been performing continuous ambulatory peritoneal dialysis for six months before having an inadvertent dialysis catheter disconnection.

**Fig. 1:**
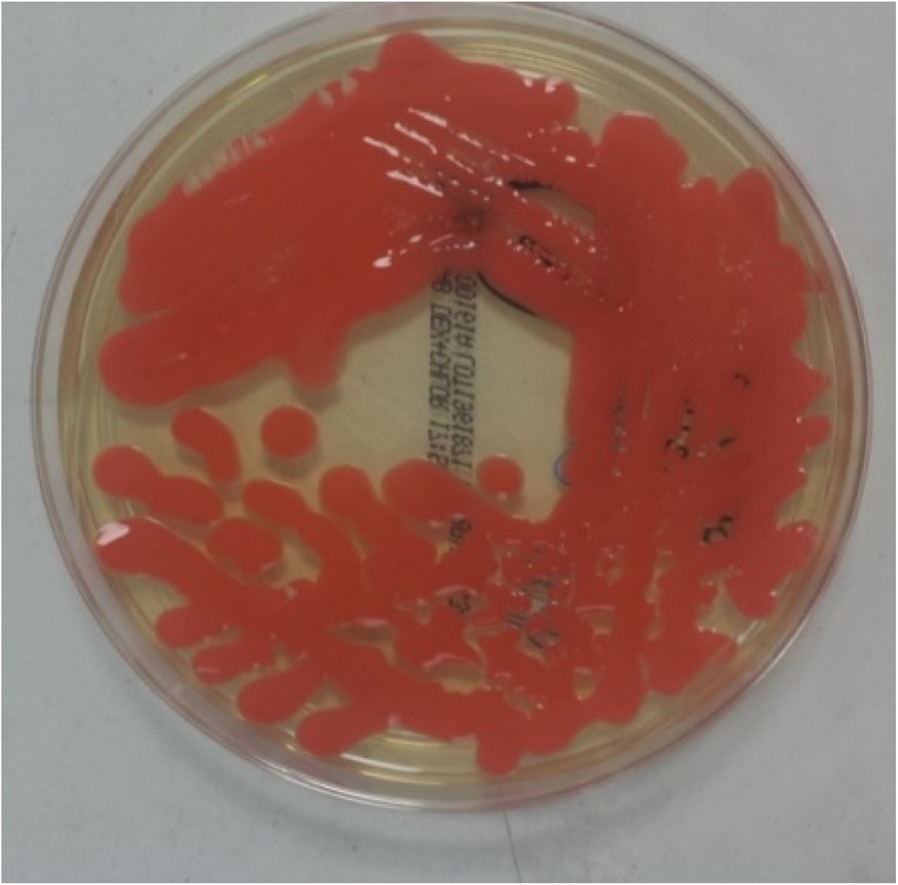
Shiny salmon-pink *Rhodotorula* colonies on Sabouraud medium

As per unit protocol he underwent a set change with concurrent prophylactic IP vancomycin administration. A sample of peritoneal dialysis fluid was sent for microscopy and culture. Initial microscopy demonstrated a white cell count of only 10 × 10^6^/l, this count was not raised therefore a differentiated cell count was not performed. No organisms were seen on Gram stain. He was clinically well with no abdominal pain, fevers or cloudy bags. The cultured peritoneal dialysis fluid grew *Rhodotorula muciliginosa* (Fig. 1). A further two peritoneal fluid samples sent up to 7 days after the first sample also grew the same *Rhodotorula spp.* suggesting colonisation. Sensitivity tests were performed at the Mycology Reference Laboratory in Bristol using microtitre broth dilution according to CLSI M-27-A3 protocol. This test showed our isolate had MIC levels of 0.25 mg/L for amphotericin, 0.50 mg/L for posoconazole and 4 mg/L for voriconazole. Our isolate was considered sensitive to amphotericin only. The patient’s C-reactive protein peaked at 30 mg/l (normal range 0-5 mg/l) with a mildly elevated white cell count of 13.8 × 10^9^/L comprised of 11.8 × 10^9^/L neutrophils and 0.8 × 10^9^/L lymphocytes. On microbiology advice, given the potential for the patient to develop fungal peritonitis, he was admitted for removal of his peritoneal dialysis catheter and commencement of IV amphotericin.

**Fig. 2:**
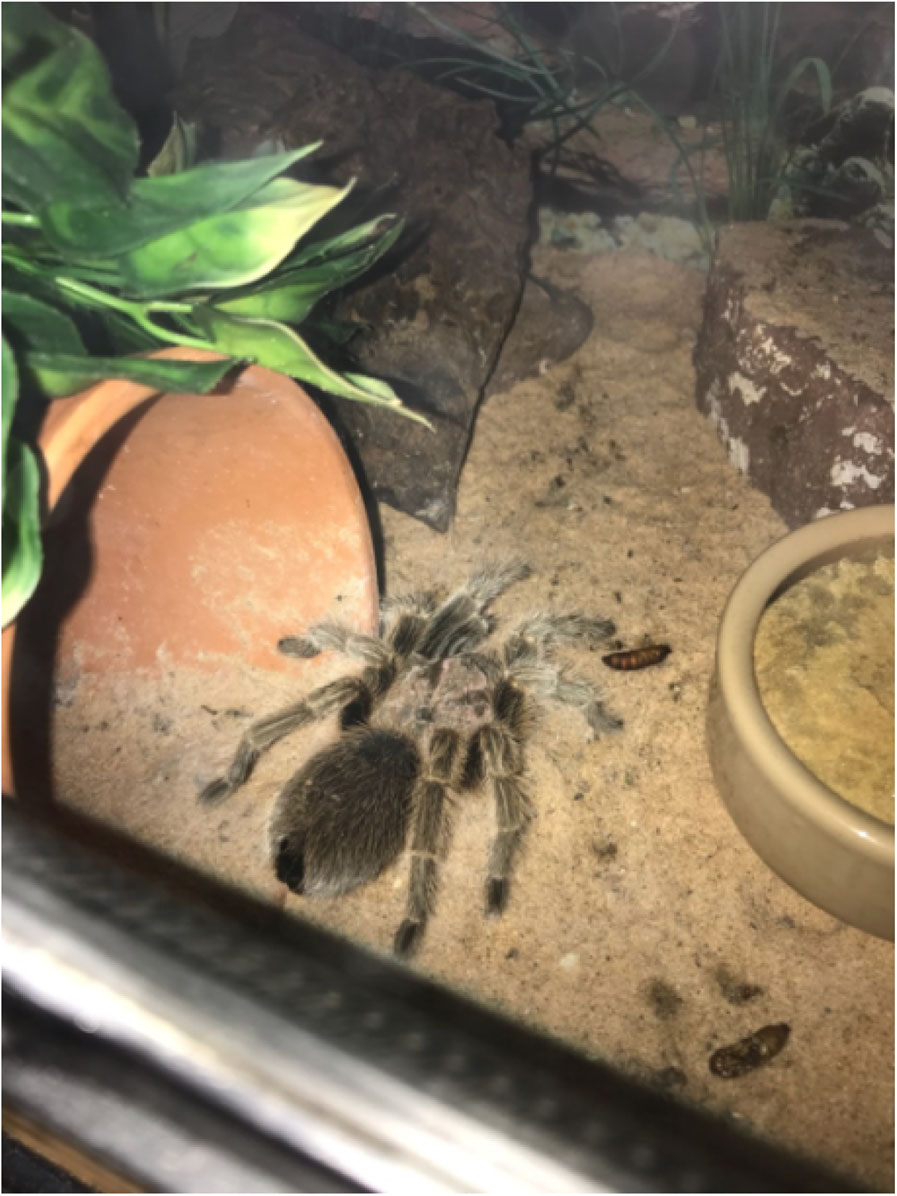
Chilean Rose Tarantula kept in the room where peritoneal dialysis exchanges occurred

An investigation into the potential source of the *Rhodotorula* spp. colonisation was commenced. It was noted that he kept a Chilean rose tarantula (Fig. 2), an Indian ornamental tarantula, and a bearded dragon, in the room where he performed his dialysis.

Samples were sent from the sand and soil environments within the animal habitats. Microbiological culture of the sand from the tarantula case grew *Rhodotorula muciliginosa*, identifying the likely source. Interestingly, *Candida spp.* was also isolated from sand from the bearded dragon case, which can also cause devastating peritonitis.

Given our patients preserved urine output, he was able to remain off renal replacement therapy for two months following completion of the Amphotericin course. The patient was pleased that dialysis could be held, and peritoneal dialysis recommenced now a source had been identified, as he wished to avoid haemodialysis. A further Tenchkoff catheter was inserted and dialysis resumed. In the interim, the cases housing the tarantulas and bearded dragon were moved to another area of the house, and a thorough deep clean of his room completed.

## Discussion and conclusions

We describe the first case where *Rhodotorula muciliginosa* has been isolated in peritoneal dialysate fluid and the likely source, the cage of a Chilean rose tarantula, identified. Zoonotic infections in humans originating from tarantulas are rare. If they occur it is usually a result of tarantula setae (hairs) penetrating the skin or eyes, or following a bite. Mechanical irritation or hypersensitivity reactions are more likely to occur rather than true infection [14]. A literature search revealed just one case of *Aspergillus* infection following ocular injury from setae in a dog [15]. Studies on the carriage of saprophytic fungi by arthropods, including spiders, has demonstrated a 40% carriage rate for *Aspergillus spp.* however *Rhodotorula spp.* was not isolated [16]*.*

Fungal infections are becoming an increasing healthcare burden. Unfortunately the number of causative pathogens, particularly affecting immunocompromised patients are growing, without an expansion in treatment options. This point was highlighted by Giacobino et al. who presented their fungal peritonitis data from a Brazilian hospital. They demonstrated causative organisms arising from a diverse group of yeasts and more importantly a variation in susceptibility to antifungals, and a significant resistance to fluconazole, causing increased difficulty in treatment [17]. The overall rates of reported *Rhodotorula spp*. infections are increasing, this is likely due to an increasing number of immunocompromised patients as well as advances in medicine resulting in more transplantation, more extensive CVC use and increasing use of broad spectrum antibiotics [4]. *Rhodotorula spp.* fungaemia has been associated with a crude mortality of 20% [18]. This figure is of particular concern with *Rhodotorula spp*. demonstrating resistance to first and second line generation triazoles, only intermediate sensitivity to amphotericin and only full sensitivity to flucytosine. As *Rhodotorula spp.* is rare the clinical breakpoint for each anti-fungal has not been published [19]**.**

Amphotericin B was used in all the reported cases of *Rhodotorula spp.* peritonitis, our report being the only case where the source was identified. Amphotericin B demonstrates concentration-dependant fungicidal activity versus candida, with a ratio of the maximum serum concentration to MIC (C_max_/MIC) of 2.4 or higher giving optimal efficacy [20]. The maximum concentration of liposomal amphotericin in peritoneal fluid to MIC (PF_max_/MIC) is probably a better correlate of efficacy when treating PD infections. Amphotericin B is 90% protein-bound and diffuses poorly into the peritoneal fluid, with correspondingly low PF_max_ levels. The more commonly used liposomal formulation has even more limited peritoneal fluid penetration and peritoneal levels may indeed be sub-therapeutic, as summarised in Table 1. In our case, the MIC of *Rhodotorula muciliginosa* was 0.25 mg/L, giving a ratio of PF_max_/MIC of between 0.5 and 3, where the optimal level is 2.4. The efficacy of liposomal amphotericin might therefore be in doubt, although our patient responded to liposomal amphotericin along with catheter removal. Intra-peritoneal administration of amphotericin is no longer recommended due to peritoneal irritation and fibrosis [7].

**Table 1:** Peritoneal concentrations of Amphotericin B [20–24]

Author and Year	Number in study	Antifungal Agent	Serum concentration (mg/L)	Peritoneal concentration (mg/L)	Peritoneal/serum penetration ratio
Polak 1979 [21]	NR	Amphotericin B	0.4 – 1.7	0.22 – 0.32	19–55%
Petersen 1978 [22]	1	Amphotericin B	0.52 (predose) 1.5 (postdose)	0.44 (predose) 0.78 (postdose)	85% 52%
Van der Voort 2007 [23]	21	Amphotericin B	0.25 (median)	0.12 (median)	41%
Weiler 2008 [20]	1	Liposomal amphotericin	NR	0.258	NR

*NR* Not reported

The significance of *Rhodotorula spp*. as a pathogenic organism in the renal population is not isolated to peritoneal dialysis as the organism has also been isolated from central venous catheters [25], suggesting the haemodialysis population could also be at risk.

The current ISPD guidelines suggest that in cases of fungal peritonitis prompt catheter removal probably improves outcome and reduces mortality with anti-fungal agents continued after catheter removal for at least 2 weeks [26]. Research suggests permanent transfer to haemodialysis is frequent following an episode of fungal peritonitis although a return to peritoneal dialysis is possible, particularly in less fragile patients. [2]. Although our patient did not meet the criteria for peritonitis the severe morbidity and mortality associated with fungal peritonitis lead us to treat the patients’ colonisation in the same manner. The ISPD guidelines also suggest a home visit by a PD nurse to detect problems with exchange technique, adherence to protocols, and to identify other environmental and behaviour issues that increase the risk of infections such as pets be carried out [26].

With unusual animal species becoming ever more popular as household pets the awareness of these as a source of infection is key. In our case, theoretically the bearded dragons case, which isolated *Candida*, was also a potential source. A good understanding of the patient’s background, home environment, and the potential for animals to be a source for colonisation and peritonitis is vital to reduce the risk of morbidity and mortality, particularly in dialysis patients.

## Data Availability

Not applicable.
